# Impacts of ZnO as a nanofertilizer on fenugreek: some biochemical parameters and SCoT analysis

**DOI:** 10.1186/s43141-023-00501-0

**Published:** 2023-05-01

**Authors:** Doaa E. Elsherif, Eman Abd-ElShafy, Asmaa M. Khalifa

**Affiliations:** 1 Botany Department, Faculty of Science, Tanta University, Tanta, 31527 Egypt; 2 Botany and Microbiology Department, Faculty of Science, Al-Azhar University (Girls Branch), Cairo, Egypt

**Keywords:** Fenugreek, Nanoparticles, MDA, DNA, SCoT analysis

## Abstract

**Background:**

Zinc oxide nanoparticles (ZnO NPs) can be considered as nanofertilizer providing zinc as an essential micronutrient for plant growth and production at specific safe dose, however, above this dose; ZnO NPs induce oxidative stress. The present research aimed to evaluate some physiological and molecular effects of ZnO NPs on *Trigonella foenum-graecum* (fenugreek) plant.

**Results:**

The ZnO NPs were applied at five different concentrations (10, 20, 30, 40, and 50 mg/l) via soaking fenugreek seeds for 24 h. Fenugreek seedlings were harvested after 14 days for biomass and biochemical analyses. The results revealed that increasing ZnO NPs concentration led to a significant increase in all measured parameters until peaked at 30 mg/l; after that, a decline trend was detected. However, malondialdehyde (MDA) increased significantly just at higher concentrations of ZnO NPs (40 and 50 mg/l). In addition, genetic variation measure using start codon targeted (SCoT) markers revealed that ZnO NP treatments exhibited limited genetic variation.

**Conclusion:**

Results showed that treatment with ZnO NPs at 30 mg/l can improve biomass, bioactive compounds, and antioxidant activity of fenugreek seedlings, besides being safe for DNA. So, this concentration could be a decent nanofertilizer for fenugreek plant.

**Supplementary Information:**

The online version contains supplementary material available at 10.1186/s43141-023-00501-0.

## Background

Application of micronutrients in the form of nanoparticles has attracted a lot of attention because of their distinctive characteristics and promising applications in many agricultural sectors [1]. Nanoparticle fertilization is an important method to release required nutrients in a controlled manner gradually, which is vital to alleviate the consequences of soil contamination generated by the excessive use of chemical fertilizers [2].

Zinc oxide (ZnO) is one of the most important nanoparticles due to its interesting and unique properties, biocompatibility, and low toxicity [3]. Many research articles studied the key role of ZnO NPs in crop growth and productivity, including nitrogen uptake, respiration, and photosynthesis, in addition to the activation of other physiological processes such as enzyme activation, synthesis of protein, and metabolism of nucleic acid and carbohydrate [4–7]. Plant response to ZnO NPs is controlled by many factors such as NPs concentration, size, exposure duration, and type of plant [8].

In contrast to the beneficial role of NPs in the aforementioned biochemical processes, the most common negative effect of NP exposure is the development of a cascade of reactions causing plants’ oxidative stress. This is due to the synthesis of high levels of reactive oxygen species (ROS) including superoxide anion (O_2_^**−**^), hydrogen superoxide (OH·), peroxide oxygen (H_2_O_2_), and singlet oxygen (“O_2_”). ROS induced-oxidative stress is triggered by activating various biochemical reactions in the plant such as lipid peroxidation (LPO) [9]. The main product of LPO is malondialdehyde (MDA) that results from the oxidation of unsaturated fatty acids on the cell membrane [10]. Moreover, NP-induced ROS may cause DNA damage via affecting cross-linking, DNA-strand breakage, and sugar or base adducts [11, 12].

Recently, many new promising marker techniques, such as targeted start codon (SCoT) polymorphism, have been used to evaluate the molecular changes in plants exposed to NPs. SCoT is reproducible marker that originates from the short-conserved region in plant genes surrounding the initiation codon of ATG translation [13]. SCoT markers have been widely used for investigation of genetic diversity and structure, identification of cultivar, quantitative trait loci (QTL) mapping, and DNA fingerprinting in plants [14]. In addition, SCoT is preferable than RAPD, ISSR, and AFLP in being more stable, providing more repeatable and reliable bands and could be used well for genetic mapping in numerous plants and marker-assisted selection programs [15].

Fenugreek (*Trigonella foenum-graecum*) is herbaceous plant whose seeds and leaves are of widespread use in food preparations and traditional medicine [16]. It is a rich source of iron, zinc, calcium, carotene, vitamin C, and many vitamins [17]. In addition, extracts of the fenugreek seeds are characterized by high phenolic acids and flavonoid contents exhibiting antioxidant activity [18]. Moreover, fenugreek seeds contain vital bioactive ingredients such as coumarin, folic acid, nicotinic acid, phytic acid, scopoletin, saponin, and trigonelline which have various therapeutic and medicinal properties [19].

This study aims to assess positive or negative influences of different concentrations of ZnO NPs on biomass, some biochemical and molecular characteristics of fenugreek plant.

## Materials and methods

### ZnO NPs characteristics

ZnO NPs were synthesized using the chemical bath deposition (CBD) method as described by El-Shaer et al. [20]. The synthesis was performed using 0.25-M zinc nitrate hexahydrate and 2.13 M of potassium hydroxide as precursors in 20 ml of deionized water. Each solution was separately stirred for 10 min and then mixed and stirred again for 10 min. The final mixture was kept in the oil bath at 80 °C for 4 h. After that, the precipitated ZnO NPs were rinsed several times with deionized water and ethanol and then dried at 105 °C. Characterization of the synthesized ZnO nanostructures, using XRD (Shimadzu 6000), UV–Vis spectrophotometer (JASCO V-630), and scanning electron microscope (JSM-651OLV) was reported in El-Shaer et al. [20] and Gaafar et al. [21]. Accordingly, the average size of the synthesized ZnO NPs was 20–45 nm.

### Plant material and growth conditions

Seeds of fenugreek (*Trigonella foenum*-*graecum* L.) Giza 30 were obtained from the Agriculture Research Centre (ARC), Ministry of Agriculture and Land Reclamation, Egypt. Seeds were disinfected with 20% of Clorox for 10 min and rinsed thoroughly to remove the disinfectant and soaked in ZnO NP concentrations (10, 20, 30, 40, and 50 mg/l) for 24 h. The same number of seeds (20 seeds per 9 cm Petri dishes) was soaked in the dark at 24–26 °C. Fenugreek seeds were then transferred in pots filled with clay and sand with ratio of 2:1. The controlled sets were also carried out at the same time along with treated seeds. The experiment was conducted with three replicas, and each pot contained 20 plants. After 14 days, the seedlings were harvested for the following growth parameters, biochemical and genetic analysis. The growth of control and treated fenugreek seedling was described using fresh and dry weights (g).

### Determination of biochemical parameters

#### Total soluble carbohydrates

In this study, total carbohydrate content was determined by the phenol–sulfuric acid method [22]. The concentration of total soluble carbohydrates content was measured at 490 nm in spectrophotometer (V-1200). It was calculated as mg/g dry weight.

#### Total soluble protein

Total soluble protein content was determined in borate buffer extract according to the method described by Bradford [23] using spectrophotometer. The concentration of total soluble protein content was calculated as mg/g dry weight.

#### Total phenolic content

The total phenolic content of fenugreek was estimated quantitatively using the method described by Jindal and Singh [24]. One milliliter of the ethanolic extract was mixed with 0.1 ml of folin reagent and 1 ml of Na_2_CO_3_ (20%) and then completed up to a known volume (5 ml) with distilled water. Thereafter, the absorbance was measured with the UV spectrophotometer, at 650 nm after 30 min. A standard curve was performed by using different concentrations of gallic acid for the determination of the total phenolic content (mg/g d.wt).

#### Total flavonoids content

Total flavonoids content was extracted by soaking 0.1 g of the dried plant in 10 ml of 95% ethanol in a water bath at 60 °C for 4 h. The clear supernatants were diluted to a known volume (10 ml). The method of aluminum chloride colorimetric was used for total flavonoids estimation [25]. The 0.5 mL of extract solution was mixed with1.5 ml of 95% ethanol, 0.1 ml of 10% aluminum chloride, 0.1 ml of 1-M potassium acetate, and 2.8 ml of distilled water. The mixture was incubated at room temperature for 30 min followed by calculation the absorbance of mixture at 415 nm using the UV spectrophotometer. The calibration curve was plotted using quercetin as a flavonoids standard. The total flavonoids concentration was expressed as mg/g d.wt.

### DPPH free radical scavenging assay

The 2,2-diphenyl-1-picrylhydrazyl (DPPH) free radical scavenging assay of CNMs was performed according to Rikabad et al. [26]. The absorbance was read at 517 nm with the same spectrophotometer.

### Glutathione analysis

The definition was based on the interaction of reduced glutathione (GSH) with DTNBA to form a yellow-colored 2-nitro5-thiobenzoate anion. The increase in the concentration of the yellow anion during this reaction was recorded spectrophotometrically at 412 nm [27]. The total glutathione content in the samples was measured (color reaction) due to forming a complex of 5,5′-dithiobis-2-nitrobenzoic acid (DTNBA) and GSH. The concentration of GSH in test samples was calculated using standard curve.

### Malondialdehyde (MDA) content

Malondialdehyde (MDA), which is a secondary end product of polyunsaturated fatty acid oxidation, was applied as an indicator of lipid peroxidation. MDA content was determined by the thiobarbituric acid (TBA) reaction as described by Heath and Packer [28]. The absorbance was measured at 532 nm and 600 nm; finally, MDA content was determined using an extinction coefficient of 1.55 m M·cm^−1^ and expressed as (n.mol/g.f.wt).

### Statistical analysis

The results were presented as mean of the replicates ± standard error (SE). Differences between treatments for the different measured variables were tested by one-way variance (ANOVA), followed by Student’s *t*-test, and Dunnett’s test with significant differences was found (*P* < 0.05) in JMP program (13.2.0).

### SCoT-PCR analysis

Genomic DNA was isolated from freshly leaves by DNeasy plant mini kit (bio basic). Moreover, using electrophoresis in 1% agarose gel with ethidium bromide, a qualitative check for DNA samples was done. Amplification reactions for six primer of SCoT techniques (Table 1) were performed as described by Fathi et al. [29] and Xiong et al. [30], respectively, and were carried out in Techne TC-512 thermal cycler as follows: one cycle at 94 °C for 4 min followed by 40 cycles of 1 min at 94 °C, 1 min at annealing temperature 57 °C for 2 min at 72 °C, and followed by 72° C for 10 min; the reaction was finally stored at 4 °C.

**Table 1:** The sequence of primer set used for SCoT analysis

No	Primer	Sequence
1	SCoT 1	F: 5′-ACG ACA TGG-3′ R: 5′-CGA CCA CGC-3′
2	SCoT 3	F: 5′-ACG ACA TGG-3′ R: 5′-CGA CCC ACA-3′
3	SCoT 4	F: 5′-ACC ATG GCT-3′ R: 5′-ACC ACC GCA-3′
4	SCoT 8	F: 5′-ACA ATG GCT-3′ R: 5′-ACC ACT GAG-3′
5	SCoT 12	F: 5′-CAA CAA TGG-3′ R: 5′-CTA CCA CCG-3′
6	SCoT 15	F: 5′-CCA TGG CTA-3′ R: 5′-CCA CCG GCT-3′

### Gel reading and analysis

Amplified products were loaded and separated on a 1.5% agarose gel with ethidium bromide and 100-bp to 3-kb ladder markers. The run was carried out for about 30 min at 100 V in mini submarine gel BioRad. DNA banding pattern photos were photographed using Bio-1D Gel Documentation system and were analyzed by GelAnalyzer3 software which scoring clear amplicons as present (1) or absent (0) for each primer and entered in the form of a binary data matrix. From this matrix, DNA profiles were performed for SCoT techniques according to Adhikari et al. [31].

## Results

### Fresh and dry weights

**Fig. 1:**
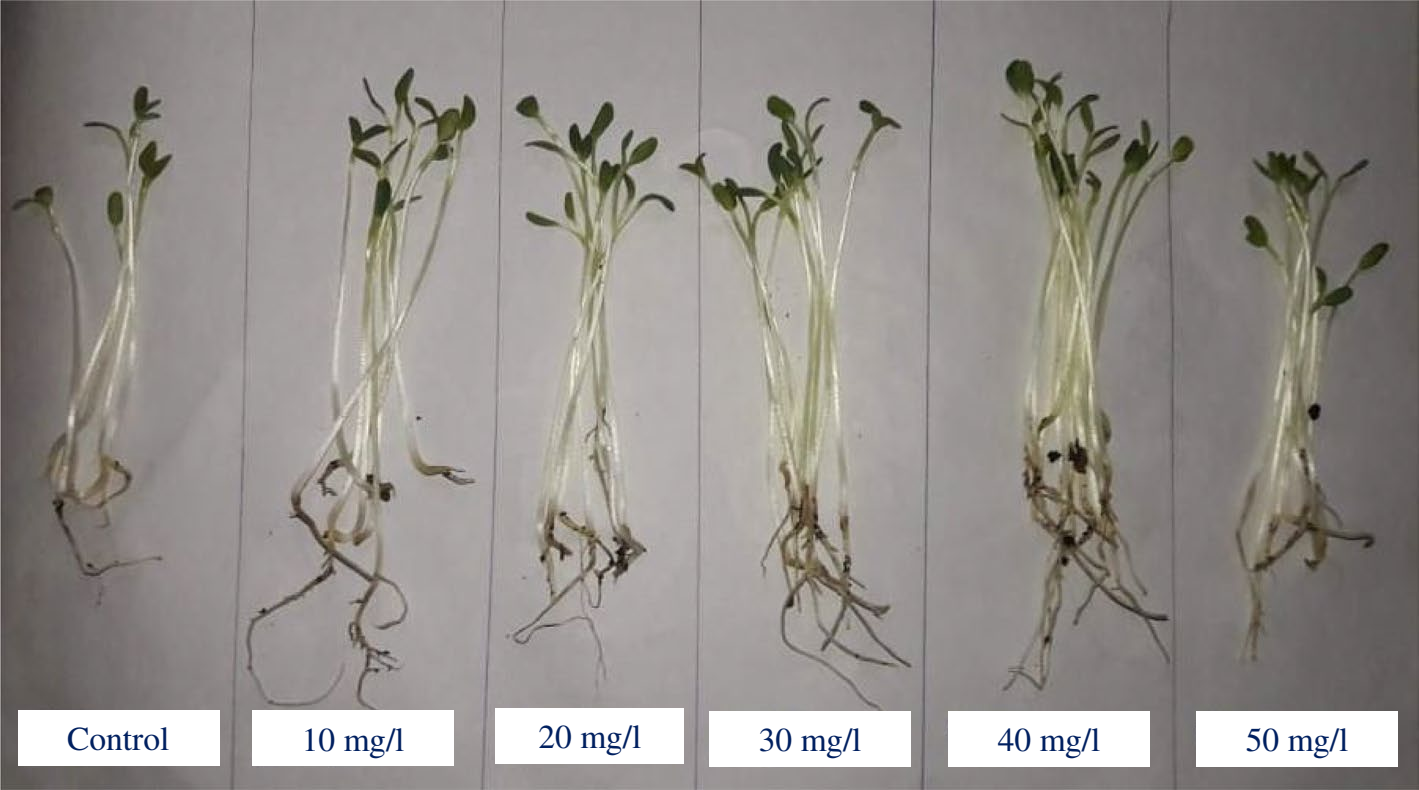
A 14-day growth of fenugreek seedlings under different concentrations of ZnO NPs

**Fig. 2:**
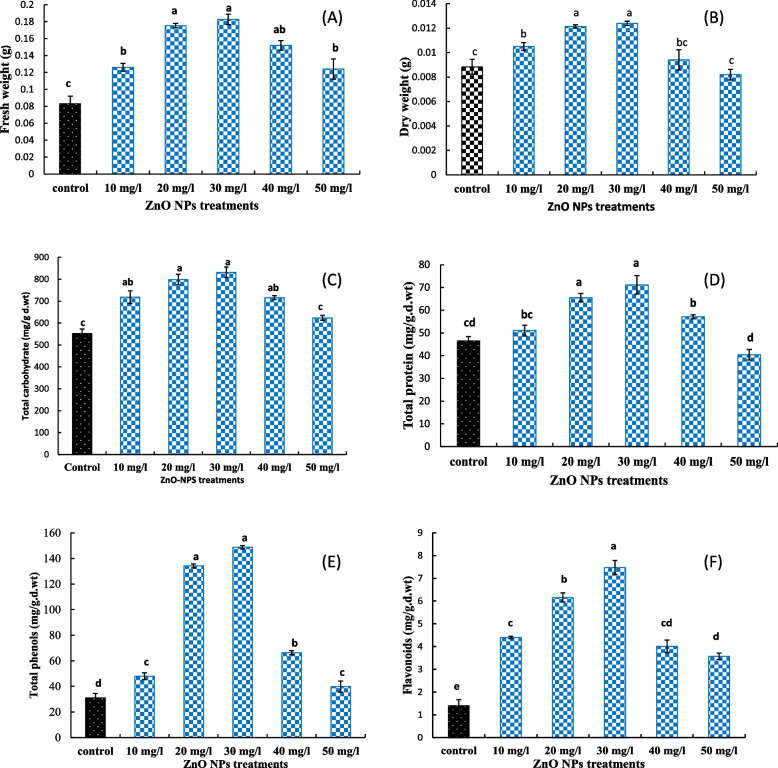
**A** Fresh weight, **B** dry weight, **C** total soluble carbohydrates, **D** total soluble protein and **E** total phenolic content, and **F** total flavonoids content of fenugreek seedling grown under different concentrations of ZnO NPs (10, 20, 30 40, and 50 mg/l). The data are means ± SE. Different small letters indicate statistically significant differences between different treatments according to the Dunnett’s test (*P* < 0.05)

The results revealed that treatment with ZnO NPs at 20 and 30 mg/l was the most effective, increasing significantly both fresh weight (0.175 ± 0.002 and 0.182 ± 0.006 g, respectively) and dry weight (0.0121 ± 0.0001 and 0.0124 ± 0.0001 g, respectively) of fenugreek seedlings, compared to control. In contrast, The ZnO NPs at 50 mg/l non-significantly reduced dry weight to 0.0082 ± 0.0004 g with respect to the control 0.0088 ± 0.0006 g (Figs. 1 and 2).

### Total soluble carbohydrates and total soluble protein

The carbohydrates content significantly increased with increasing ZnO NPs concentration, until reached maximum at 30 mg/l ZnO NPs; after that, a decline trend was detected but still higher than the control (Fig. 2). Regarding the total protein content, a similar increasing then declining trend was achieved with increasing ZnO NPs concentration, except at 50 mg/l that non-significantly decreased relative to the control. Treatment with 30 mg/l ZnO NPs was the most effective, and increased carbohydrate and protein contents by 50.6 and 52.8%, respectively, over the control (Fig. 2).

### Total phenolic content and total flavonoids content

The same trend observed for total carbohydrate and protein was also detected for total phenolic and total flavonoids contents. They increased significantly, with respect to the control, with increasing ZnO NPs concentration, until peaked at 30 mg/l ZnO NPs, after that a decreased trend was detected but still significantly higher than the control. Also, treatment with ZnO NPs at 30 mg/l was still the most effective, increasing phenolic to 148.79 ± 1.33 mg/g.d.wt and flavonoids to 7.48 ± 0.306 mg/g.d.wt compared to the control (30.95 ± 3.64, 1.43 ± 0.23 mg/g.d.wt, respectively) (Fig. 2).

### DPPH radical antioxidant activity

**Fig. 3:**
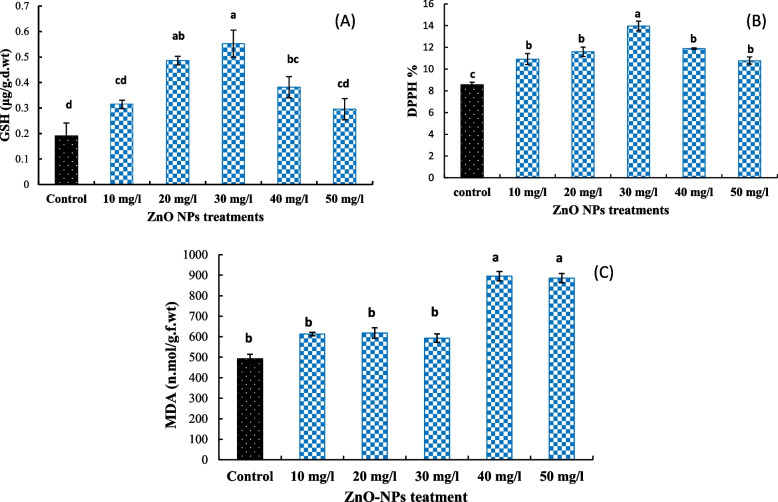
**A** GSH content, **B** DPPH radical antioxidant activity, and **C** MDA content under different concentrations of ZnO NPs (10, 20, 30 40, and 50 mg/l). The data are means ± SE. Different small letters indicate statistically significant differences between different treatments according to the Dunnett’s test (*P* < 0.05)

The effect of different concentrations of ZnO NPs on DPPH radical antioxidant activity is shown in Fig. 3. Our results revealed that all ZnO NPs tested concentrations significantly increased DPPH radical antioxidant activity relative to the control, with nonsignificant difference between 10, 20, 40, and 50 mg/l ZnO NPs treatments. Treatment with ZnO NPs at 30 mg/l exhibited the highest DPPH activity increasing by 13.9% over the untreated control.

### GSH content

As shown in Fig. 3, ZnO NPs treatments upregulated GSH level at all treated concentrations compared to the control. This increase started to be significant at 20 mg/l (155% increase than the control) and reached maximum at 30 mg/l which motivated the highest GSH level (190% increase than the control). The doses of 10 and 50 mg/l ZnO NPs non-significantly upregulated GSH content of fenugreek seedlings (65 and 55% increases over the control, respectively).

### MDA content

In order to evaluate the membrane damage imposed by ZnO NPs, MDA content (Fig. 3) was measured to analyze lipid peroxidation. Only treatments at 40 and 50 mg/l of ZnO NPs significantly increased MDA content by 81.6 and 79.7%, respectively, over the control, while no significant increases in plants treated with 10, 20, or 30 mg/l of ZnO NPs when compared with the control.

### SCoT analysis

**Fig. 4:**
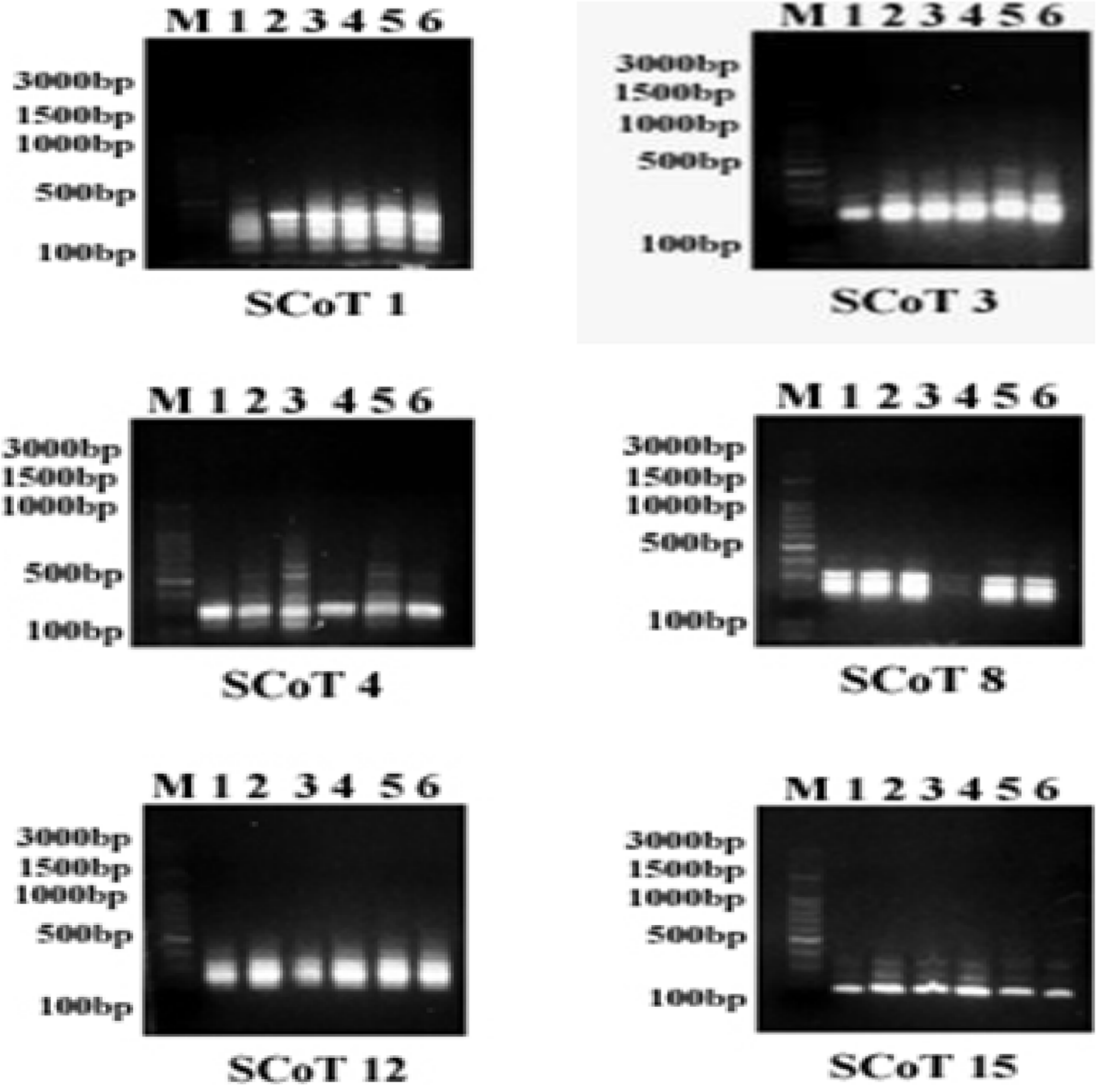
The SCoT marker created with various six primers (SCoT 1, SCoT 3, SCoT 4, SCoT 8, SCoT 12, SCoT15) to detect the impact of ZnO NPs in fenugreek seedlings. Lanes: M, DNA ladder; 1, control; and 2–6, ZnO NPs doses (10, 20, 30, 40, and 50 mg/l)

In SCoT analysis, a measure of genetic variation, six primers screened for amplification of all the treatments and the control. All primers gave reproducible and scorable amplification product. Table 2 showed codes of the six primers, total number of amplification fragments for control and ZnO NPs treatments, and the number of polymorphic fragments for each primer. A total of 24 bands were obtained in which 7 were polymorphic and 17 were monomorphic with a polymorphism 29.16% across the six primers (Fig. 4).

**Table 2:** SCoT analysis screened by six primers that demonstrated the effect of ZnO NPs on DNA pattern of fenugreek plant

**Primer**	**Total** **band**	**Monomorphic** **band**	**Polymorphic** **band**	**Unique** **band**	**Mo.wt** **(bP)**		**ZnO NPs (mg/l)**				
						**C**	**10**	**20**	**30**	**40**	**50**
					540	1	0	1	1	1	1
					400	1	1	1	1	1	1
**SCoT 1**	5	4	1	1	325	1	1	1	1	1	1
					185	1	1	1	1	1	1
					130	1	1	1	1	1	1
					500	0	1	1	1	1	1
**SCoT 3**	3	2	1	1	320	1	1	1	1	1	1
					245	1	1	1	1	1	1
					640	0	0	1	0	1	0
					580	1	1	1	1	1	1
**SCoT 4**	5	3	2	1	380	0	1	1	1	1	1
					300	1	1	1	1	1	1
					240	1	1	1	1	1	1
					430	1	1	1	0	1	1
					340	1	1	1	1	1	1
**SCoT 8**	4	3	1	1	275	1	1	1	1	1	1
					220	1	1	1	1	1	1
					615	0	0	0	0	0	1
					485	1	1	0	1	1	1
**SCoT 12**	4	2	2	1	370	1	1	1	1	1	1
					280	1	1	1	1	1	1
					360	1	1	1	1	1	1
**SCoT 15**	3	3	-	-	245	1	1	1	1	1	1
					185	1	1	1	1	1	1
**Total**	**24**	**17**	**7**	**5**							

*Mo.wt* ladder molecular weight, *bp* base pair, *C* control

In primer SCoT 3, a band at 500 bp was detected for all ZnO NPs treated plants in contrast to the control. Also, SCoT 4 revealed a band at 380 bp for all ZnO NPs treatments, but it was absent in the control.

In the case of SCoT 8, a band at 430 bp was observed in the control and all treatments except 30 mg/l ZnO NPs, while primer SCoT12 detected one unique band at 615 bp in 50 mg/l ZnO NPs treatment.

## Discussion

Nanoparticles can be used as plant fertilizers to make nutrients bioavailability in a controlled manner so that they are only absorbed by the plant and are not lost to the surrounding environment including soil, water, and associated microorganisms [32]. Zinc oxide nanofertilizers exhibited preferable and promising results in enhancing seed germination and promoting healthy seedlings [33].

Our findings showed that the soaking of fenugreek seeds in different ZnO NPs concentrations was considerably effective and promoted the growth, and this was dose dependent. Both fresh and dry weights were increased in all plants treated with different ZnO NPs concentrations, except at 50 mg/l which revealed a nonsignificant decrease in dry weight, compared to the control. This increase can be certified with the tendency of ZnO NPs to penetrate seed testa and improve the Zn use efficiency as an essential micronutrient leading to increase seedling growth [34, 35]. In agreement with our obtained results, Atteya et al. [36] and Gheith et al. [37] stated that zinc treatment promoted growth and yield parameters of jojoba and maize plants. They also observed the enhancing effect of nanoparticles for plant growth and yield in peanut at lower doses. In addition, Zn NPs were found to improve the length, leaf protein, and dry mass of Pearl Millet (*Pennisetum americanum*) plant [38, 39]. On the other hand, Liu et al. [40] stated that high concentrations of ZnO NPs can inhibit germination, biomass, and photosynthesis of plants. This may explain the decrease of dry weight at 50 mg/l Zno NPs.

The application of ZnO NPs significantly affected the assessed total proteins and total carbohydrate of fenugreek seedlings. The total soluble protein and carbohydrate contents increased simultaneously with increasing the nanoparticle dose and reached the maximum at 30 mg/l while dropped slightly following treatment with 40 and 50 mg/l ZnO NP. These findings agree with Rao et al. [41] and Zhao et al. [42] who declared that nanofertilizers have a pronounced influence on carbohydrates biosynthesis in leaves and can modify protein content of plants. In addition, the ZnO NPs at lower concentrations increased the total soluble protein and carbohydrate contents in various plant species such as cluster bean [11], green pea [43], maize [44], and bell peppers [45]. These findings are compatible with the fact that zinc at definite concentrations is vital for structural and catalytic constituents of proteins and enzymes as cofactors which are essential for normal plant growth and development [46].

Exposure of fenugreek seeds to ZnO NPs at different concentrations affected positively on the seedling contents of flavonoids and phenols, with a highest increase at 30 mg/l. Also, Uresti-Porras et al. [45] found that bell peppers treated with ZnO NPs at concentrations of 30 mg/l revealed a significant increase in total phenolic compounds over the control. In addition, Mohammadghasemi et al. [47] concluded that nanofertilizers improved total phenolic and total flavonoid contents in *Lallemantia iberica* when compared with the control. Moreover, Zn nanofertilizer was found to increase the polyphenols content in cotton and soybean crops [48, 49]. These improvements may be in line for the essential role of zinc in the carbon allocation to biosynthesize phenolic compounds in shikimic acid and acetate pathways [50].

The DPPH has been widely used to estimate the antioxidant activity of plant extracts via testing the ability of compounds to scavenge free radical or donate hydrogen [51]. In the present study, all tested ZnO NP concentrations increased the yield of total DPPH over the untreated control plants. No significant differences were observed between 10, 20, 40, and 50 mg/l Zno NPs treatments, while 30 mg/l revealed the highest DPPH activity. This agrees with Salachna et al. [52] who found that exposure to ZnO NPs at low concentration improved the total polyphenols content, antioxidant activity, and DPPH activity with respective to the control in *Perilla* (*Perilla frutescens* (L.)) plant. In addition, Thapa et al. [53] stated that the total antioxidant activity (TAA), DPPH, and flavonoid contents increased in mung bean (*Vigna radiata*) plants treated with zinc sulfide nanoparticle. Moreover, Weisany et al. [48] concluded that nanofertilizers improve the antioxidant capacity and DPPH scavenging in rice.

Glutathione content increased in all treatments of ZnO NPs and peaked at 30 mg/l compared with the control. This agrees with Riaz et al. [54] who found that SiNPs increased glutathione content in wheat. In addition, Jurkow et al. [55] found that foliar application of Au-NPs and Pt-NPs on oakleaf lettuce seedlings leads to an increase in glutathione (GSH) content. Moreover, the genes involved in glutathione biosynthetic were upregulated in *Arabidopsis thaliana* plants treated with 0.2–1 mg/l Ag-NPs [56].

Lipid peroxidation acts as a symptom of membrane degradation under stress conditions and is positively related with MDA content [57]. In this study, the content of MDA, as an oxidative stress index, in plants treated with 10, 20, or 30 mg/l of ZnO NPs did not significantly differ from the control. However, the higher concentrations of ZnO NPs, 40 and 50 mg/l, significantly increased MDA content. Also, Singh et al. [58] stated that ZnO NPs at higher concentration provoked the ROS production causing oxidative damage that increases MDA content in chickpea plants. In spite of the vital role of zinc in protection and stabilization of the biological membranes against integrity loss and permeability alteration and oxidative stress, the higher doses of ZnO NPs may damage this membrane via enhancing ROS mechanism that produces oxidative stress [59].

SCoT markers were implemented to detect the genomic changes that occurred in the fenugreek seedling upon exposure to different doses of ZnO NPs. The SCoT banding patterns pertaining to the impact of ZnO NPs show limited genetic variations between the ZnO NPs treatments and the control plants. A possible reason for these limited genomic changes in ZnO NPs-treated plants could be attributed to lower levels of ZnO NPs that had been tested.

## Conclusion

Our results collectively reflect that soaking of fenugreek seeds in 30 mg/l ZnO NPs was found to be the most effective nanofertilizer between tested concentrations. The 30 mg/l ZnO NPs attained the highest values for plant biomass, flavonoid content, phenolic content, and antioxidant activity in fenugreek seedling, giving rise to a potential increase in the nutraceutical properties of fenugreek plant. SCoT markers revealed that treatment of fenugreek plant with ZnO NPs until 50 mg/l did not exhibit distinct DNA alterations.

## Supplementary Information

**Additional file 1. Table 3.** The impact of different concentrations of ZnO NPs on biomass and biochemical constituents of fenugreek plants.

## Data Availability

All the data required for review article is available upon request through the corresponding author.
