# Supplementary material for: Impacts of ZnO as a nanofertilizer on fenugreek: some biochemical parameters and SCoT analysis
Source: J Genet Eng Biotechnol. 2023 May 1;21:52. doi: 10.1186/s43141-023-00501-0 (PMC10151287; doi:10.1186/s43141-023-00501-0)
Supplement: Supplementary file 1 — Additional file 1. Table 3. The impact of different concentrations of ZnO NPs on biomass and biochemical constituents of fenugreek plants. [file 43141_2023_501_MOESM1_ESM.docx]

**Table 3.**The impact of different concentrations of ZnO NPs on biomass and biochemical constituents of fenugreek plants

| **Zn NPs (mg/l)** | **Fresh wt.**  **(g)** | **Dry wt.**  **(g)** | **Protein**  **(mg/g d.wt)** | **Carbohydrate**  **(mg/g d.wt)** | **Phenols**  **(mg/g d.wt )** | **Flavonoids**  **(mg/g d.wt)** | **DPPH**  **(%)** | **MDA**  **(n.mol/g.f.wt)** | **GSH**  **(μg/g.d.wt)** |
| --- | --- | --- | --- | --- | --- | --- | --- | --- | --- |
| **Control** | 0.083^c^±0.008 | 0.0088^c^±0.0006 | 46.55^cd^±1.75 | 551.28^c^±20.68 | 30.95^d^±3.64 | 1.43^e^±0.23 | 8.55^c^±0.231 | 492.90^b^±21.26 | 0.190^d^±0.050 |
| **10** | 0.126^b^±.004 | 0.010^b^±0.0003 | 51.03^bc^±2.36 | 717.89^ab^±29.27 | 49.58^c^±10.80 | 4.4^c^±0.055 | 10.92^b^±0.514 | 612.62^b^±8.13 | 0.314^cd^±0.016 |
| **20** | 0.175^a^±0.002 | 0.0121^a^±0.0001 | 65.52^a^±1.83 | 798.75^a^±24.14 | 134.22^a^±1.57 | 6.16^b^±0.198 | 11.59^b^±0.427 | 618.45^b^±25.64 | 0.485^ab^±0.016 |
| **30** | 0.182^a^±0.006 | 0.0124^a^±0.0001 | 71.14^a^±4.02 | 830.60^a^±23.94 | 148.79^a^±1.33 | 7.48^a^±0.306 | 13.96^a^±0.455 | 592.87^b^±20.46 | 0.552^a^±0.053 |
| **40** | 0.152^ab^ ±0.005 | 0.009^bc^±0.0008 | 57.09^b^±0.87 | 800.22^ab^±8.83 | 66.32^b^±1.64 | 4.01^cd^±0.275 | 11.88^b^±0.064 | 895.12^a^±23.25 | 0.380^bc^±0.041 |
| **50** | 0.124 ^b^ ±0.012 | 0.0082^c^±0.0004 | 46.55^cd^±2.32 | 681.14^c^±10.68 | 39.79^cd^±4.34 | 3.57^d^±0.145 | 10.77^b^±0.333 | 885.82^a^±22.42 | 0.295^cd^±0.041 |
